# Machine Learning to Predict Risk of Relapse Using Cytologic Image Markers in Patients With Acute Myeloid Leukemia Posthematopoietic Cell Transplantation

**DOI:** 10.1200/CCI.21.00156

**Published:** 2022-05-06

**Authors:** Sara Arabyarmohammadi, Patrick Leo, Vidya Sankar Viswanathan, Andrew Janowczyk, German Corredor, Pingfu Fu, Howard Meyerson, Leland Metheny, Anant Madabhushi

**Affiliations:** ^1^ Department of Computer and Data Sciences, Case Western Reserve University, Cleveland, OH; ^2^ Department of Biomedical Engineering, Case Western Reserve University, Cleveland, OH; ^3^ Lausanne University Hospital, Precision Oncology Center, Vaud, Switzerland; ^4^ Department of Population and Quantitative Health Sciences, School of Medicine, Case Western Reserve University, Cleveland, OH; ^5^ Department of Pathology, University Hospitals Cleveland Medical Center, Cleveland, OH; ^6^ Department of Hematology and Oncology, University Hospitals Cleveland Medical Center, Cleveland, OH; ^7^ Louis Stokes Veterans Administration Medical Center, Cleveland, OH

## Abstract

Allogenic hematopoietic stem-cell transplant (HCT) is a curative therapy for acute myeloid leukemia (AML) and myelodysplastic syndrome (MDS). Relapse post-HCT is the most common cause of treatment failure and is associated with a poor prognosis. Pathologist-based visual assessment of aspirate images and the manual myeloblast counting have shown to be predictive of relapse post-HCT. However, this approach is time-intensive and subjective. The premise of this study was to explore whether computer-extracted morphology and texture features from myeloblasts' chromatin patterns could help predict relapse and prognosticate relapse-free survival (RFS) after HCT.

**MATERIALS AND METHODS:**

In this study, Wright-Giemsa–stained post-HCT aspirate images were collected from 92 patients with AML/MDS who were randomly assigned into a training set (*S*_*t*_ = 52) and a validation set (*S*_*v*_ = 40). First, a deep learning–based model was developed to segment myeloblasts. A total of 214 texture and shape descriptors were then extracted from the segmented myeloblasts on aspirate slide images. A risk score on the basis of texture features of myeloblast chromatin patterns was generated by using the least absolute shrinkage and selection operator with a Cox regression model.

**RESULTS:**

The risk score was associated with RFS in *S*_*t*_ (hazard ratio = 2.38; 95% CI, 1.4 to 3.95; *P* = .0008) and *S*_*v*_ (hazard ratio = 1.57; 95% CI, 1.01 to 2.45; *P* = .044). We also demonstrate that this resulting signature was predictive of AML relapse with an area under the receiver operating characteristic curve of 0.71 within *S*_*v*_. All the relevant code is available at GitHub.

**CONCLUSION:**

The texture features extracted from chromatin patterns of myeloblasts can predict post-HCT relapse and prognosticate RFS of patients with AML/MDS.

## INTRODUCTION

Myelodysplastic syndromes (MDSs) and acute myeloid leukemia (AML)^1^ are hematologic diseases that are challenging to treat because of the associated high morbidity and mortality along with high rates of relapse. MDS constitutes a group of clonal hematopoietic disorders characterized by ineffective hematopoiesis and peripheral blood cytopenias. MDS can be either indolent or quickly progressive with a high risk of transformation into AML.^2,3^ AML is defined by the infiltration of bone marrow or peripheral blood by > 20% myeloblasts, commonly referred to as blasts, which do not undergo the typical lineage-specific WBC differentiation.^4^ Consequently, these blasts overtake healthy stem cells in the blood and bone marrow.^2,5^ As a result, patients with AML experience infection, anemia, and poor blood clotting.^6^ Thus, detecting and quantifying myeloblasts plays an important role in diagnosing and monitoring response to treatment in AML.^7^

**CONTEXT**

**Key Objective**
Allogenic hematopoietic stem-cell transplant (HCT) is a last-resort therapy for acute myeloid leukemia (AML) that has a poor prognosis. Predicting relapse post-HCT could help direct more aggressive treatment to those patients who need it. In this study, we explore machine learning–extracted texture features from bone marrow aspirate slide images to predict relapse and to prognosticate relapse-free survival post-HCT.
**Knowledge Generated**
The machine learning model helped to identify unique morphologic and texture differences within the myeloblasts of the bone marrow aspirate images of patients with AML who were at a higher risk of relapse post-HCT.
**Relevance**
After prospective validation, the new machine classifier presented in this study could enable risk stratification of patients with AML, helping to identify patients who would relapse from those who would not within 5 years of HCT.

Nearly 40%-60% of patients with AML who achieve complete remission eventually relapse unless given consolidation therapy. Allogenic hematopoietic stem-cell transplantation (HCT), a procedure where a human leukocyte antigen matched or partially matched donor's hematopoietic system replaces the recipient hematopoietic system after immunosuppressive therapy, is the best postremission consolidation therapy. HCT is often the only curative option for patients with high-risk AML; however, it is associated with significant morbidity and mortality because of graft-versus-host disease and immunosuppression.^2-6^ Although relapse rates can be reduced by intensifying conditioning chemotherapy, there is a concomitant increase in treatment-related mortality.^6,8^ When patients relapse, the prognosis is especially poor, more so in the setting of early relapse where the patient cannot endure further intensive chemotherapy.^9,10^ Moreover, only a minority of relapsed patients improve with salvage therapies such as donor lymphocyte infusions or a second HCT in selected patients.^9,10^. The low response rate, poor improvement under salvage therapy, and substantial side effects of these treatments make it vital to direct them only to patients at high risk of AML relapse.

An increase in the bone marrow blast percentage heralds the relapse of AML. The gold standard for diagnosis of relapse post-transplant is review of aspirates from bone marrow biopsy to evaluate blast percentage and morphology. In this process, a pathologist counts approximately 200-300 cells from randomly chosen regions of a bone marrow aspirate specimen, and if 5% or more of the cells are blasts, the patient is considered to have relapsed.^11^ The examination of only a small proportion of cells and the limits of human perception contribute to limiting the accuracy and reproducibility of this process.^11-14^ In this study, we are interested in using an automated computational approach to go beyond the pathologist-based visual assessment to identify features associated with relapse post-HCT and relapse-free survival (RFS). A reproducible and objective image analysis approach could allow for the segmentation of myeloblasts and subsequent extraction of subvisual features potentially carrying prognostic and predictive information regarding relapse post-HCT.

There have been many studies on using computational image analysis for disease prognosis from digitized histologic images of solid tumors.^15,16^ However, there is limited literature in cytopathology, with most studies focusing on cell segmentation, WBC classification, and automated cell counting^17-20^ rather than outcome prediction and prognosis.

**FIG 1.**
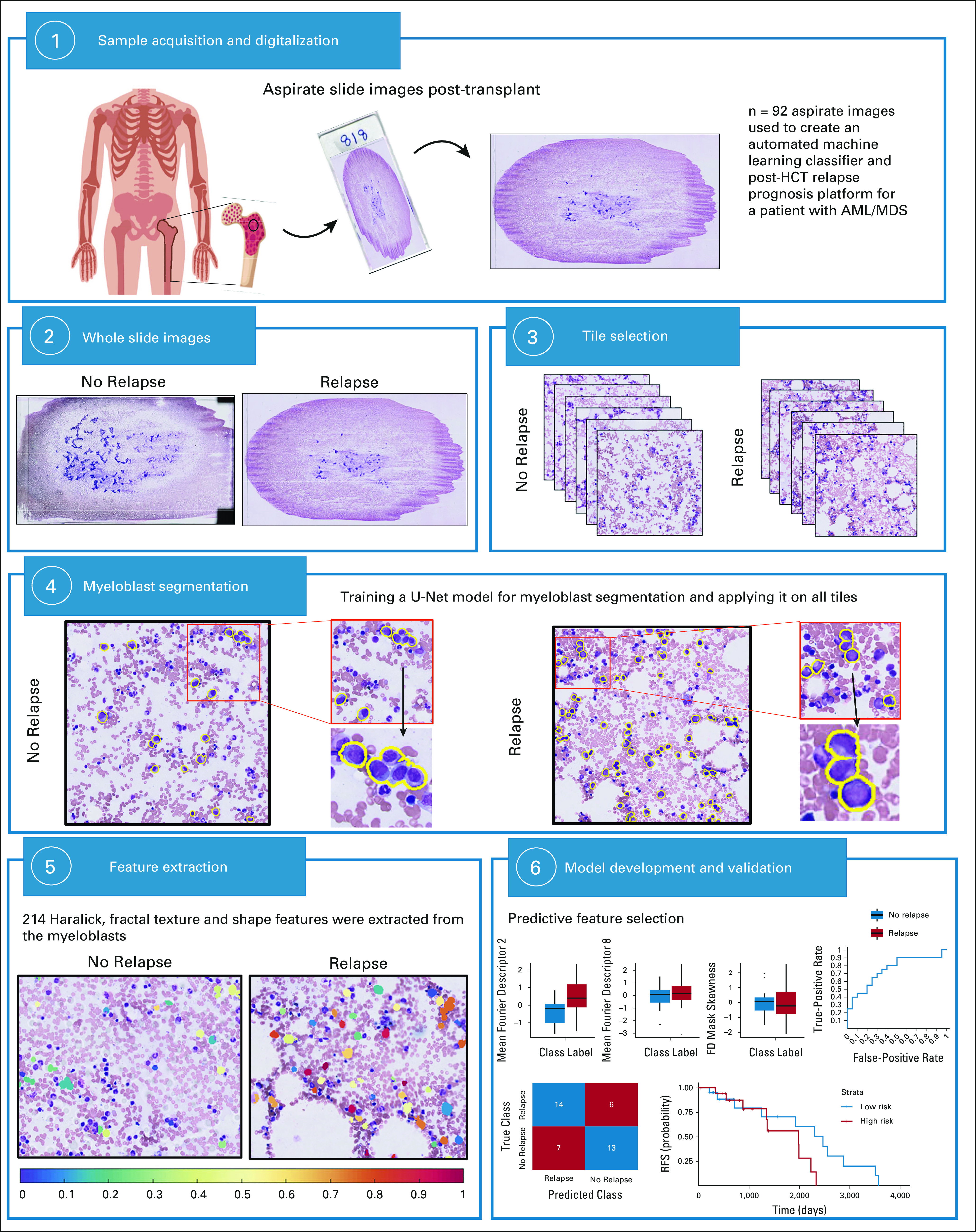
Overview of the approach used in this article. First, the data set was randomly divided into training (*S*_*t*_, n = 52) and validation (*S*_*v*_, n = 40) sets. Six random 512 × 512 micron tiles were then selected from every Wright-Giemsa–stained aspirate slide image. Myeloblasts were segmented on all tiles, and features associated with the myeloblast shape and chromatic pattern were extracted. A subset of two features (contrast variance and correlation skewness) most correlating with relapse in the training data set were identified. Using these features, a LDA model for PRS was derived using *S*_*t*_. This PRS was locked down and then validated on *S*_*v*_. This figure has been designed using resources from Freepik.com.^21^ AML, acute myeloid leukemia; HCT, hematopoietic stem-cell transplant; LDA, linear discriminant analysis; MDS, myelodysplastic syndrome; PRS, pathological risk score; RFS, relapse-free survival.

In this study, we aimed to construct a quantitative pathological risk score (PRS) that uses features from myeloblasts derived from routine Wright-Giemsa–stained bone marrow aspirate images to (1) prognosticate RFS and (2) predict relapse post-HCT for patients with AML/MDS (see Fig 1). Our approach used a novel model for myeloblast segmentation and used computational methods to analyze the quantity, texture, and shape of myeloblasts. Using a cohort of 92 patients treated after HCT, obtained from the University Hospitals Cleveland Medical Center (UH), we extracted image features from myeloblasts and identified a subset of features in post-HCT patients (1) associated with RFS and (2) predictive of relapse.

## MATERIALS AND METHODS

### Patient Selection

**FIG 2.**
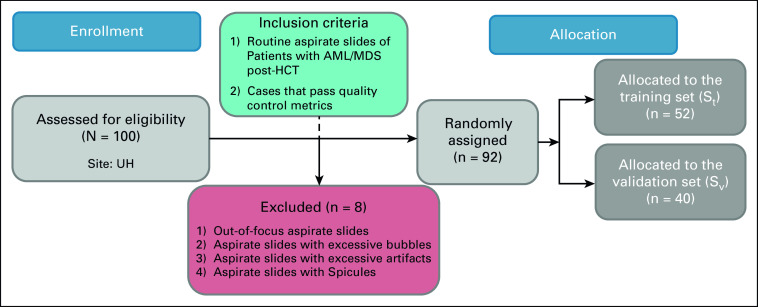
A CONSORT diagram outlining the eligibility criteria and distribution of patients in this study. AML, acute myeloid leukemia; HCT, hematopoietic stem-cell transplant; MDS, myelodysplastic syndrome; UH, University Hospitals Cleveland Medical Center.

Under an institutional review board–approved protocol, a chart review was performed to identify patients with AML or MDS who underwent HCT between January 1, 2009, and January 1, 2020, at the University Hospitals Cleveland Medical Center. Wright-Giemsa–stained bone marrow aspirate slides from 92 patients with AML/MDS (see Fig 2) were collected 6-8 weeks after HCT. Of these patients, 48 had a relapse of AML within the first year. All slides were digitized at 40× magnification. Six random nonoverlapping 512 × 512 micron (2048 × 2048 pixel) tiles were selected from regions within each digitized aspirate slide image with dense WBCs and no artifacts, or bubbles, for a total of 552 tiles. Patients were randomly divided into training (*S*_*t*_, n = 40 with 20 relapsed) and validation sets (*S*_*v*_, n = 52 with 28 relapsed). Patients who did not experience relapse were censored at the date of the last follow-up.

RFS was defined as the time interval between the start of treatment (date of HCT) and the date of relapse, or the date of death whichever occurred earlier, in patients with AML. For censored patients, the survival is defined between the HCT date and the last follow-up date.

### Image Analysis

#### Blast detection and segmentation.

A blast segmentation framework on the basis of u-net,^22^ a type of deep learning architecture, was trained on 795 64 × 64 micron (256 × 256 pixel) patches from 35 patients annotated for myeloblasts by a hematopathologist. Of these, 79 random patches were held out for model testing. On the held-out test set, the model yielded a per-pixel true-positive rate of 0.99, a true-negative rate of 0.96, and an F1 score of 0.76. Segmentation was then performed on all 552 tiles from 92 aspirate slide images, and results were visually verified to be suitable for feature extraction.

#### Feature extraction.

Features designed to reflect chromatin patterns, heterogeneity, shape complexity, and shape irregularity were extracted from each segmented myeloblast (see Table 1). The mean, median, standard deviation, and skewness of each feature were calculated across all myeloblasts on every tile from a patient to arrive at a tile-level feature value and again across all six tiles to produce a patient-level value. This process yielded a 214-feature vector (see the Data Supplement) for each patient, which encodes their associated blast presentation characteristics.

**TABLE 1.**
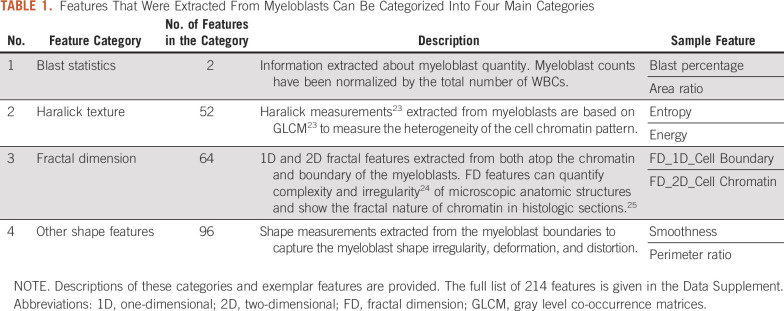
Features That Were Extracted From Myeloblasts Can Be Categorized Into Four Main Categories

### Model Construction and Statistical Analysis

#### Feature selection.

The least absolute shrinkage and selection operator (LASSO) from the glmnet package in R^26^ was used in an internal cross-validation fashion on *S*_*t*_ to tune the lambda (λ) parameter. This process selected two texture features (ie, average of contrast variance and average of correlation skewness) from the 214 features as most relevant for prognosticating RFS. Additional details about LASSO are provided in the Data Supplement. For convenience, we denote $S_{\hat{t}}$ and $S_{\hat{v}}$ as the subsets of the feature spaces of *S*_*t*_ and *S*_*v*_ containing these two texture features.

#### Prediction of relapse.

A linear discriminant analysis (LDA) classifier was trained on $S_{\hat{t}}$ to predict which patients would experience relapse post-HCT therapy. The ability to identify relapse post-HCT was assessed by the area under the receiver operating characteristic curve (AUC) in $S_{\hat{v}}$. Accuracy, sensitivity, and specificity were also computed at the optimal operating point of the receiver operating characteristic curve (ROC), defined as the threshold that maximized overall accuracy.

#### Prognostic model creation and evaluation.

$S_{\hat{t}}$ was subsequently used to construct a Cox proportional hazards model to obtain the PRS for each patient. Model performance was evaluated by the Kaplan-Meier method, the log-rank test, the hazard ratio (HR; 95% CI), and Harrell's concordance index (C index [95% CI]). Mean PRS in $S_{\hat{t}}$ was used as a threshold in both $S_{\hat{t}}$ and $S_{\hat{v}}$ to dichotomize patients into high-risk/low-risk categories.

#### Myeloblast baseline.

To evaluate the effectiveness of machine-based myeloblast percentage alone in predicting relapse, segmented myeloblasts from all tiles for each patient were counted and were normalized by the total number of WBCs for each patient. This feature was used to train a LDA classifier to predict relapse and was also used to prognosticate RFS. A comparison was then performed between machine-based myeloblast counting and machine-detected texture features in predicting relapse and prognosticating RFS.

### Ethics Approval

This study (STUDY IRB NUMBER 20210380) was conducted in full accordance with the Health Insurance Portability and Accountability Act (HIPAA) regulations after approval from the Institutional Review Board (IRB) at Case Western Reserve University (Cleveland, OH). The IRB waived the requirements for informed consent of all patients because of the retrospective, non-interventional, and non-therapeutic nature of this study.

## RESULTS

### Patient Characteristics

The characteristics of the patients used in this study are summarized in Table 2. Among the 48 relapse patients, the median time to relapse was 269 (range: 47-1,574) days, with 60% of these relapses occurring within 1 year of HCT. Among the 12 patients who relapsed beyond 18 months, the median time to relapse was 2.3 (range: 1.7-4.3) years.

**TABLE 2.**
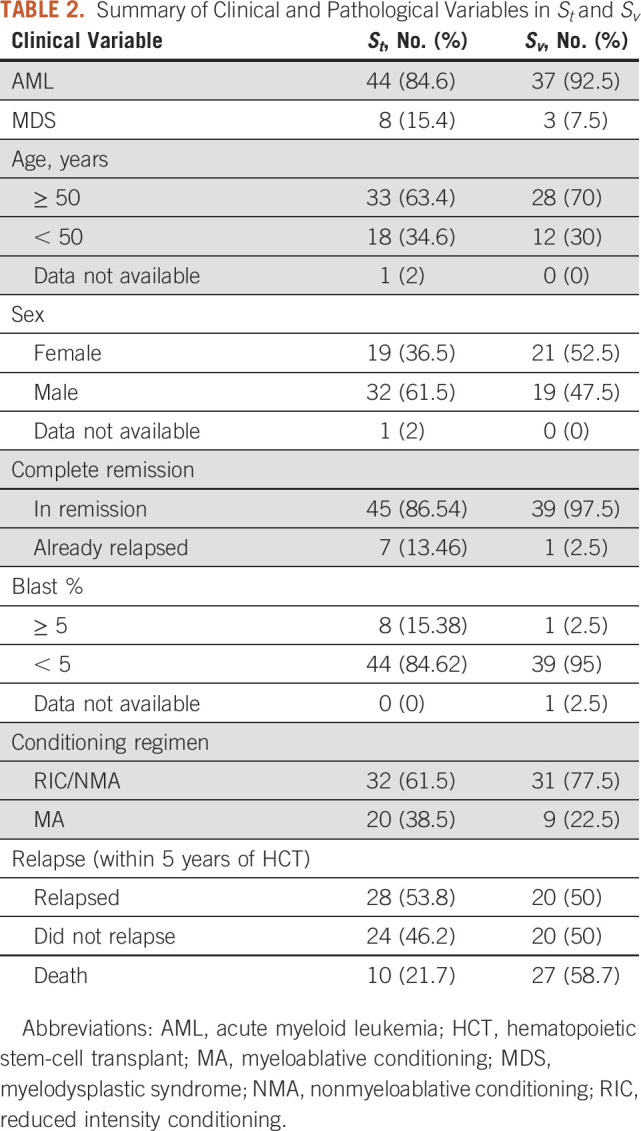
Summary of Clinical and Pathological Variables in *S*_*t*_ and *S*_*v*_

### Experiment 1: Myeloblast Texture Features Are Associated With AML Relapse Post-HCT

LASSO was used for feature selection in *S*_*t*_, and the Haralick texture features of *contrast variance* and *correlation skewness* were selected from 214 features to form the PRS. Both contrast variance and correlation skewness were reflecting the differences in chromatin patterns of the myeloblasts.^27^ The texture feature of image contrast indicates the large differences between neighboring pixels, whereas the image correlation mostly focuses on the similarity of pixels and gives a low weight to elements with dissimilar gray levels.^28^ These two features were subsequently used to build the LDA classifier for predicting relapse post-HCT. In $S_{\hat{v}}$, this classifier was able to distinguish relapse from no-relapse patients with an AUC of 0.71, an accuracy of 0.68, a sensitivity of 0.8, and a precision of 0.64.

**FIG 3.**
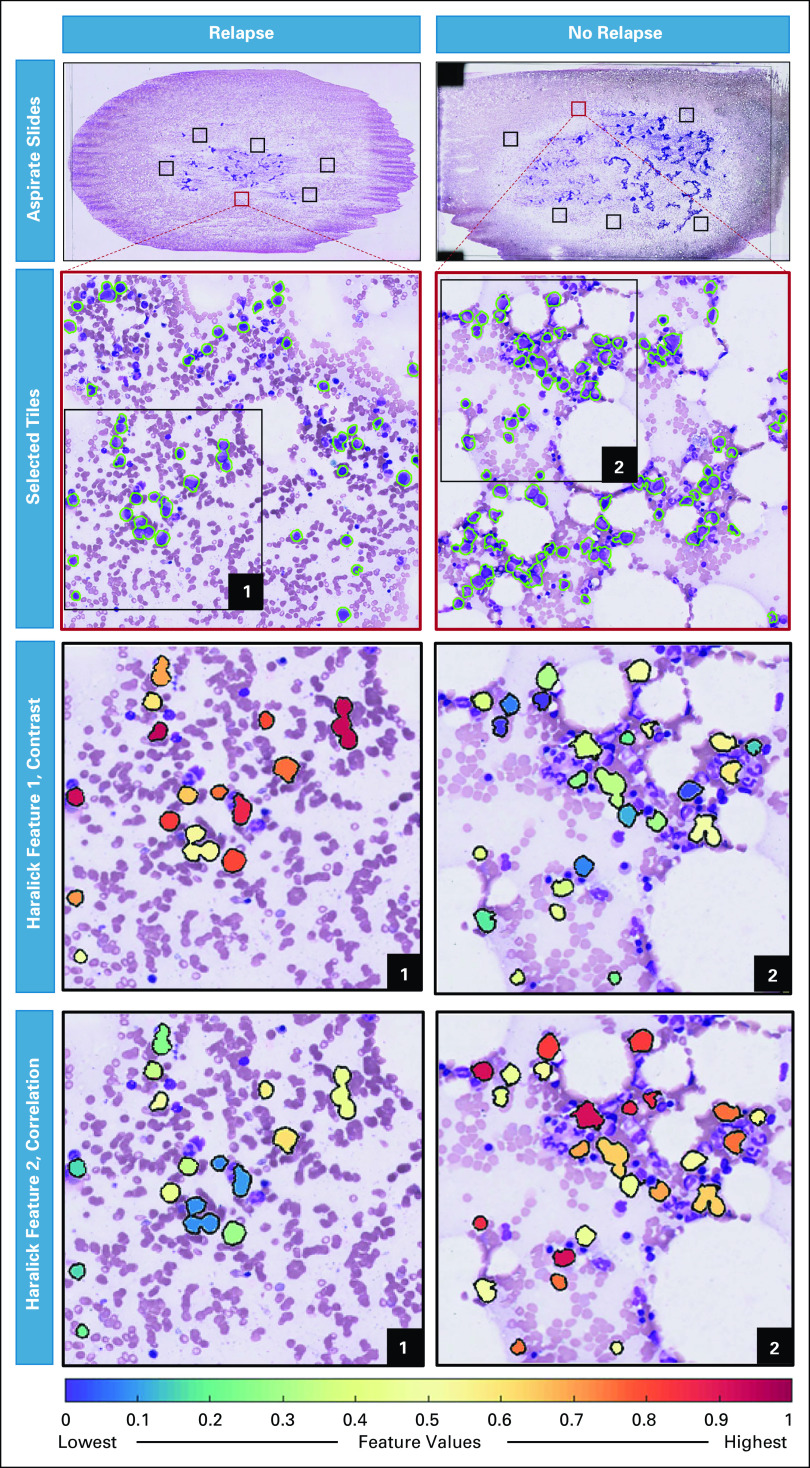
The two texture features of contrast and correlation that construct PRS visualized for patients experiencing relapse and no relapse. The Haralick contrast feature appears to have higher values in relapse patients compared with no-relapse patients. Conversely, the Haralick correlation feature has higher values in no-relapse patients on average. PRS, pathological risk score.

Qualitatively, Figure 3 illustrates the discriminability of the myeloblast's contrast and correlation features for representative no-relapse and relapse patients. There is higher textural pattern disorder (ie, heterogeneity) within myeloblasts of a relapse patient for Haralick contrast feature. Lower values were observed within myeloblasts of a relapse patient for Haralick correlation feature as compared with myeloblasts of a no-relapse patient.

These results highlight that the contrast and correlation can begin to predict HCT outcomes (relapse versus no-relapse) when used within a LDA classifier.

### Experiment 2: Myeloblast Texture Features Are Associated With RFS in AML/MDS

**FIG 4.**
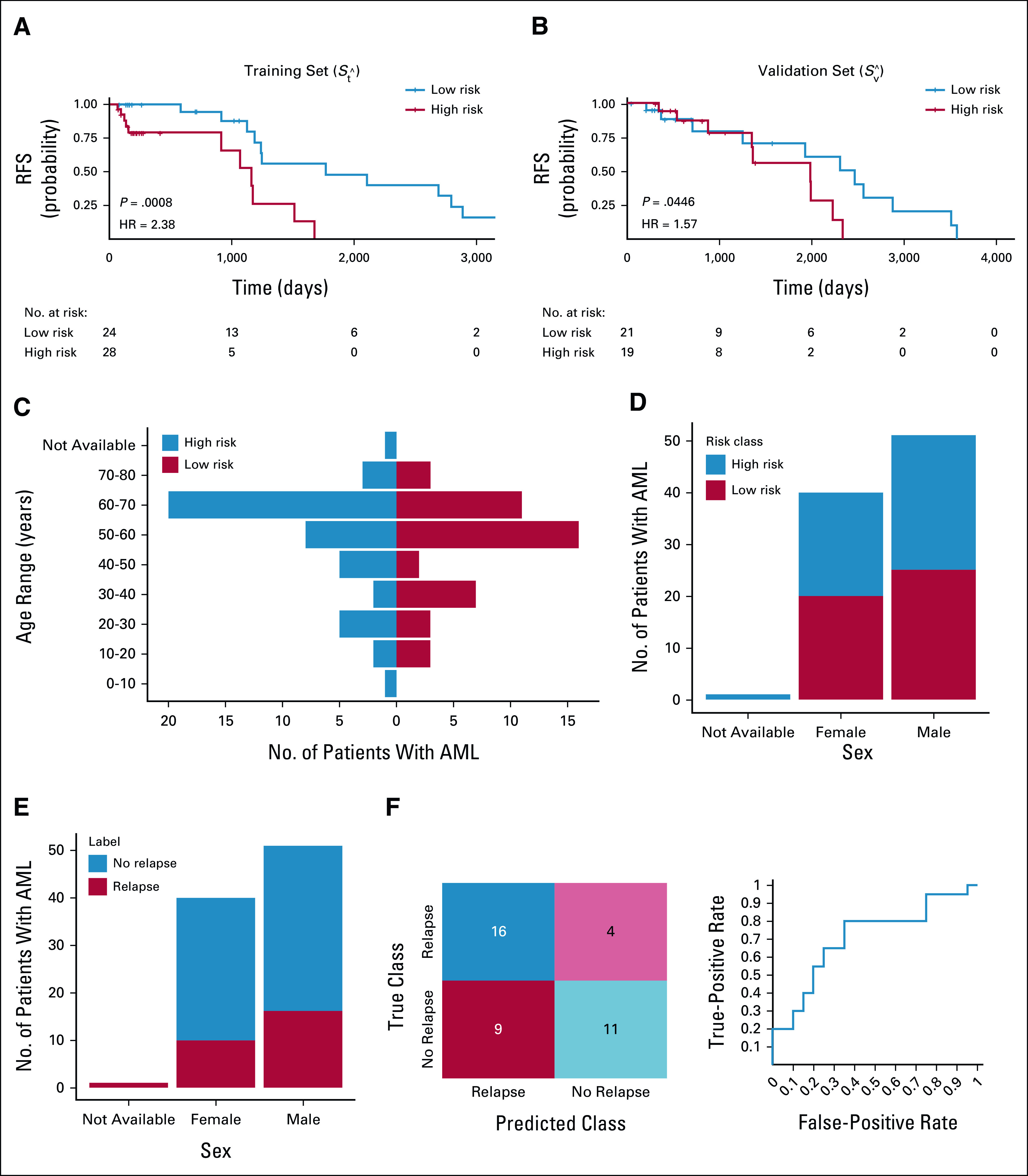
The Kaplan-Meier curves of the high-risk (red) and low-risk groups (blue) in (A) $S_{\hat{t}}$ (training set; HR = 2.38, 95% CI, 1.43 to 3:95; *P* = .0008) and (B) $S_{\hat{v}}$ (validation set; HR = 1.58; 95% CI, 1.01 to 2.4; *P* = .04); (C) distribution of high-risk and low-risk patients in different age ranges, with (D) and (E) showing the sex distribution in different groups; and (F) the LDA classification results via both a confusion matrix and the ROC curve. AML, acute myeloid leukemia; HR, hazard ratio; LDA, linear discriminant analysis; ROC, receiver operating characteristic curve.

A univariate Cox regression analysis developed using the contrast variance and correlation skewness features indicated that PRS was significantly negatively associated with RFS in both $S_{\hat{t}}$ (HR = 2.38; 95% CI, 1.43 to 3.95; *P* = .0008) and $S_{\hat{v}}$ (HR = 1.58; 95% CI, 1.01 to 2.45; *P* = .04). The corresponding Kaplan-Meier survival curves (see Fig 4) show a significant difference in RFS between patients with low and high PRS ($S_{\hat{t}}$: *P* = .0008, $S_{\hat{v}}$: *P* = .04).

A multivariable Cox regression model indicated that PRS was the only biomarker associated with RFS in *S*_*t*_ (PRS: HR = 3.09; 95% CI, 1.52 to 6.27; *P* = .002; sex: HR = 0.98; 95% CI, 0.33 to 2.87; *P* = .97; age: HR = 0.99, 95% CI, 0.96 to 1.03; *P* = .85; pathologist blast percentage: HR = 0.80; 95% CI, 0.41 to 1.57; *P* = .51; conditioning regimen: HR = 0.99; 95% CI, 0.35 to 2.83; *P* = .98; comorbidity index: HR = 1.17; 95% CI, 0.89 to 1.53; *P* = .27; disease type: HR = 0.14; 95% CI, 0.01 to 1.51; *P* = .11; C-index = 0.76) and also with RFS in *S*_*v*_ (PRS: HR = 1.83; 95% CI, 1.05 to 3.20; *P* = .03; sex: HR = 1.31; 95% CI, 0.48 to 3.55; *P* = .60; age: HR = 1.01; 95% CI, 0.98 to 1.05; *P* = .37; pathologist blast percentage: HR = 0.91; 95% CI, 0.34 to 2.46; *P* = .86; conditioning regimen: HR = 0.54; 95% CI, 0.11 to 2.66; *P* = .45; comorbidity index: HR = 1.44; 95% CI, 1.06 to 1.96; *P* = .03; disease type: HR = 1.85; 95% CI, 0.58 to 5.95; *P* = .30; C-index = 0.74).

### Experiment 3: Comparison of Myeloblast Texture Features Versus Machine-Derived Myeloblast Percentage

Finally, a comparison between our classifier and the clinical standard of machine-derived blast percentage is shown in Table 3. These metrics demonstrate that our image biomarker was better able to differentiate between relapse and no-relapse patients post-HCT and were also more robust in prognosticating RFS.

**TABLE 3.**
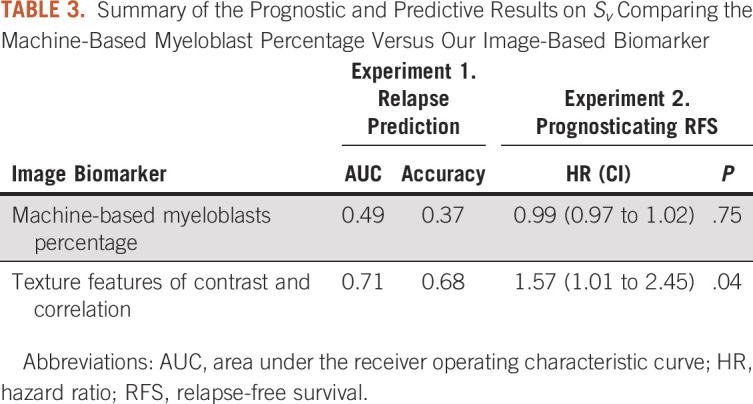
Summary of the Prognostic and Predictive Results on *S*_*v*_ Comparing the Machine-Based Myeloblast Percentage Versus Our Image-Based Biomarker

## DISCUSSION

Timely prediction of AML relapse after allogenic HCT is crucial to direct chemotherapy to high-risk patients only. Traditionally, manual counting of the myeloblasts on aspirate smear slides by hematopathologists is used to discover which patients will relapse post-HCT.^29^ However, this method is time-consuming and error-prone.^11-14^ We also know that the myeloblast count may fail to distinguish relapse patients and other approaches such as high-risk cytogenetics can better predict relapse.^29^ Aside from prognostic factors, such as relevant molecular and cytogenetic aberrations,^25^ routine analysis of cytologic images reveals crucial information on cell physiology.^13^ Our approach of computational image analysis of aspirate images goes above and beyond myeloblast count, aiming to capture myeloblast morphology and appearance. The significance of cytologic interrogation of cells in different types of leukemias has also been suggested in other studies.^11,13,14,25,30,31^ Textural and morphological differences that we measure in myeloblasts using our method offer an approximate estimate of complexity in chromatin patterns,^12,25^ which may be related to how patients respond to treatment.^14^ As an example, Auer rods or cytoplasmic granules are reddish, linear structures composed of fused primary granules that may exist in leukemic myeloblasts. Their presence indicates myeloid malignancy, which may lead to resistance to treatments or ultimately relapse.^32,33^ In addition, computational analysis of myeloblasts across aspirate images to capture information about post-HCT relapse is in consonance with the current laboratory diagnosis of hematologic disorders that are also generally based on evaluation of characteristics of blood cell chromatin patterns in peripheral blood smears and bone marrow.^34,32^ The rationale behind this diagnosis is that the chromatin pattern especially in the nucleus is related to cell function, and therefore, the abnormalities within the nucleus chromatin are associated with the malignancy.^35^ Therefore, interrogation of myeloblast shape and texture features using computational analysis would allow the development of accurate decision support tools for prognosticating relapse after transplantation.

Previous work^11,36,37^ on predicting probability of relapse in patients with AML focused on traditional visual (or manual) blast counts and clinical markers (eg, cytogenetic risk stratification). Although other studies were focused on automating and replicating a pathologist's manual review, this work aimed to explore prognostic and predictive features derived from myeloblast presentation. We studied features in the context of two AML use cases, predicting (1) post-HCT relapse and (2) RFS, and used a hand-crafted feature-engineering approach, with features designed to quantify characteristics of myeloblast cells as described by hematopathologists.^38^ These features, we hypothesize, correspond to traits of appearance and chromatin texture that are biologically known and interpretable. This contrasts with more opaque deep learning approaches where the features are extracted in an unsupervised manner and do not necessarily have an informed biologic rationale. The relative simplicity of our models stands is another advantage over deep learning approaches, which often uses models trained with millions of parameters that cannot be biologically interpreted.^39^

Results from our first experiment showed that textural features of contrast variance and correlation skewness were predictive of relapse post-HCT, with less skewed correlation between myeloblasts and more contrast variance, that is, higher texture heterogeneity^28^ being associated with increased risk of relapse. This finding is concordant with other studies, which have associated chromatin pattern heterogeneity and complexity with cytoplasmic and membranous protein expression.^13,40^ Therefore, greater heterogeneity in chromatin pattern presentation may indicate a lack of cell maturation, driving disease relapse.^13,40^ The notion that myeloblasts with higher contrast variance values are associated with elevated relapse risk is also consistent with previous studies.^41-43^ These studies found that higher heterogeneity in leukemic cells (myeloblasts) is a result of multiple mutations in the nucleus, which lead to patient resistance to therapy and relapse.^41,42,44,45^ Taken together, myeloblast chromatin patterns reflect the total sum of various underlying biologic interactions and thus may provide utility in prognostic prediction.

In addition, our results suggest these features were not only predictive of relapse but were also associated with RFS of patients with AML post-transplant. Our findings were consistent with previous work in which cell chromatin pattern heterogeneity and complexity reflected DNA methylation patterns^13,29^ and are related to patient shorter overall survival.^13,31^ Other studies found that increases in roughness of cell surfaces in patients with leukemia were associated with clinical response to therapy.^14^ These findings motivate the appearance of cells in leukemia cases as possessing information about a patient's disease-free survival post-therapy. In this study, patients with myeloblasts of smoother chromatin (lower contrast variance) texture were more likely to respond to treatment, whereas patients with higher myeloblast chromatin contrast variance (higher heterogeneity) mostly experienced AML relapse post-HCT.

In relation to existing clinical AML grading relapse and in agreement with the study by Yeung et al,^29^ we found that myeloblast count was not a good predictive or prognostic feature. This finding contributes to the growing body of work, which suggests that textural features are much more predictive of AML relapse than simple myeloblast counts.

Our study had some limitations worth noting. One was the relatively small size of the validation cohort and the fact that these came from a single institution. The study was retrospective in nature and not prospective. In addition, we did not compare the PRS against well-established clinical and cytogenetic/molecular markers such as mutations in DNMT3A and IDH,^46^ limitations we intend to address in future work. To ensure the validity of PRS for clinical use, prospective clinical trials will be needed to be performed. Patients with AML/MDS who are categorized as high risk by the PRS may merit the maintenance of treatment intensity by consistently using concurrent chemotherapy or intensifying chemotherapy. Taken together, this would represent a novel, viable precision oncology approach to treating patients who undergo HCT in the modern era.

In summary, we developed a quantitative PRS, on the basis of two features related to the textural appearance of myeloblasts, automatically extracted from bone marrow cytologic images of patients with AML. PRS was prognostic of RFS after HCT in patients with AML/MDS. A machine classifier in conjunction with the myeloblast texture parameters was able to predict relapse post-HCT. Further multisite validation including retrospective validation of archived samples from completed clinical trials followed by large prospective clinical trial evaluation is necessary to validate PRS as a prognostic and predictive biomarker to risk stratify patients post-HCT.

## Data Availability

The data underlying this article were provided by the University Hospitals Cleveland Medical Center (UH) under license/by permission. Data will be shared on request to the corresponding author with the permission of UH.
